# A Quantitative Comparison of Anti-HIV Gene Therapy Delivered to Hematopoietic Stem Cells versus CD4+ T Cells

**DOI:** 10.1371/journal.pcbi.1003681

**Published:** 2014-06-19

**Authors:** Borislav Savkovic, James Nichols, Donald Birkett, Tanya Applegate, Scott Ledger, Geoff Symonds, John M. Murray

**Affiliations:** 1 School of Mathematics and Statistics, University of New South Wales, Sydney, Australia; 2 Department of Clinical Pharmacology, Flinders University, Adelaide, Australia; 3 Kirby Institute, University of New South Wales, Sydney, Australia; 4 Faculty of Medicine, University of New South Wales, Sydney, Australia; 5 St Vincent's Centre for Applied Medical Research, Darlinghurst, New South Wales, Sydney, Australia; 6 Calimmune Pty Ltd, Darlinghurst, New South Wales, Australia; Gladstone Institute (UCSF), United States of America

## Abstract

Gene therapy represents an alternative and promising anti-HIV modality to highly active antiretroviral therapy. It involves the introduction of a protective gene into a cell, thereby conferring protection against HIV. While clinical trials to date have delivered gene therapy to CD4+T cells or to CD34+ hematopoietic stem cells (HSC), the relative benefits of each of these two cellular targets have not been conclusively determined. In the present analysis, we investigated the relative merits of delivering a dual construct (CCR5 entry inhibitor + C46 fusion inhibitor) to either CD4+T cells or to CD34+ HSC. Using mathematical modelling, we determined the impact of each scenario in terms of total CD4+T cell counts over a 10 year period, and also in terms of inhibition of CCR5 and CXCR4 tropic virus. Our modelling determined that therapy delivery to CD34+ HSC generally resulted in better outcomes than delivery to CD4+T cells. An early one-off therapy delivery to CD34+ HSC, assuming that 20% of CD34+ HSC in the bone marrow were gene-modified (G+), resulted in total CD4+T cell counts ≥180 cells/ µL in peripheral blood after 10 years. If the uninfected G+ CD4+T cells (in addition to exhibiting lower likelihood of becoming productively infected) also exhibited reduced levels of bystander apoptosis (92.5% reduction) over non gene-modified (G-) CD4+T cells, then total CD4+T cell counts of ≥350 cells/ µL were observed after 10 years, even if initially only 10% of CD34+ HSC in the bone marrow received the protective gene. Taken together our results indicate that: 1.) therapy delivery to CD34+ HSC will result in better outcomes than delivery to CD4+T cells, and 2.) a greater impact of gene therapy will be observed if G+ CD4+T cells exhibit reduced levels of bystander apoptosis over G- CD4+T cells.

## Author Summary

HIV infects and depletes the body's immune cells (CD4+T cells), and if untreated results in Acquired Immunodeficiency Syndrome (AIDS) and mortality approximately 10 years after initial infection. To protect the host against HIV induced immune depletion, either the main target cells (CD4+T cells) or the stem cells that produce the immune cells (hematopoietic stem cells) can be targeted for treatment with gene therapy. Gene therapy is the process of altering the genetic code of the host cell by the use of an integrative virus which has been modified to be safe and express the desirable genes. While a limited number of clinical studies have delivered gene therapy to either cellular target, the relative merits of each approach in terms of efficacy of AIDS treatment remain poorly understood. In the present analysis, we modelled clinical outcomes with gene therapy delivery to either CD4+T cells or to HSC. We found that delivery to HSC would result in better outcomes and the establishment of a persistent population of gene-modified CD4+T cells. These results provide important quantitative insights that may serve to optimize gene therapy delivery in upcoming clinical trials.

## Introduction

Anti-HIV gene therapy represents a promising alternative treatment to combination antiretroviral therapy (cART) [1]–[5]. It involves the introduction of a protective gene into a cell, thereby conferring protection against HIV. While cART is a life-long systemic treatment that suffers from toxicity, co-morbidity, attendant compliance and viral resistance concerns [6]–[8], gene therapy may be envisaged as a full or partial replacement for cART that may help overcome these issues. A therapy that reduces or eliminates the need for continued systemic treatment holds significant advantages.

While genetic constructs may be introduced into a cell to inhibit various stages of the HIV infection pathway [9] (including pre-entry, pre-integration, and post-integration), several lines of evidence, including predictions from mathematical modelling [10], now indicate that inhibition of viral entry is most likely toachieve best clinical outcomes. Additionally, over 95% of HIV-induced cell death has been attributed to bystander apoptosis resulting from viral entry into a cell without viral integration into the cellular genome [11]. Suppressing viral binding to the CCR5 receptor induces additional benefits. Individuals with a 32 base pair deletion in their CCR5 gene (Δ-32) have reduced CCR5 expression on the surface of their CD4+T cells, and achieve full (homozygous) or partial (heterozygous) protection against HIV infection [12]–[15]. The importance of targeting the CCR5 mode of viral entry is further supported by the “curative effect” seen from transplantation of Δ-32 mutation hematopoietic stem cells to the “Berlin patient” with AIDS and leukaemia [16]. Collectively these observations have given strong impetus for gene therapy constructs that inhibit/target the entry stage of the HIV infection cycle.

Gene therapy can be delivered to a number of cellular targets including CD4+T cells [1] and CD34+ hematopoietic stem cells (HSC) [3]. While safety and indication of biological effect in HIV-infected individuals have been observed for delivery to CD4+T cells [17]–[24] and to CD34+ HSC [25]–[29], the clinical impact of each cellular target in terms of long-term preservation of total CD4+T cell counts and forestalment of AIDS remains uncertain. The relative merits of one cellular target over the other remain poorly understood.

In the present analysis we are concerned with a quantitative comparison of the merits of delivering an anti-HIV gene therapy into either CD4+T cells or into CD34+ HSC. We consider a dual anti-HIV genetic construct containing both a CCR5 entry inhibitor [1] and a C46 fusion inhibitor [23], [30], which will be delivered in-vivo in an upcoming phase I/II clinical trial conducted by members of our group [31]. While CCR5 inhibitors employing zinc finger nucleases have recently reported high-levels of protection against HIV in humanized mice studies [32], [33] and provided an indication of therapeutic effect in the ongoing phase I/II clinical trials SB-728-T [34]–[36], it is now also well-established that blocking or down-regulating the CCR5 co-receptor favours selection for CXCR4 (X4) tropic virus [37]–[39]. The emergence of ×4 virus is of concern, since ×4 viral strains are generally associated with accelerated progression to AIDS [40]–[42]. These observations of increased ×4 selection when the CCR5 co-receptor is blocked have provided strong impetus for anti-HIV gene therapy that, in addition to a CCR5 inhibitor, also employs an additional inhibitor to suppress ×4 viral strains [23], [30].

Previously we modelled the long-term in-vivo dynamics of an anti-HIV ribozyme (OZ1) [43], that was delivered autologously to CD34+ HSC in a recent phase II clinical trial conducted by members of our group [28]. In the present analysis we extend upon this previous modelling and investigate the long-term impact of delivering a dual anti-HIV gene construct (CCR5 entry inhibitor + C46 fusion inhibitor) to either CD4+T cells or to CD34+ HSC. Gene therapy delivery would be achieved through large-volume apheresis, cell selection for either CD34+ HSC or CD4+T cells, transduction with the gene therapeutics, followed by re-infusion [18], [44]. We evaluate the likely clinical impact in terms of preservation of total CD4+T cells, as well as in terms of forestalment of AIDS. The following points of interest were also investigated for therapy delivery to each of the two cellular targets:

1. The impact if gene therapy is first administered at early, intermediate or late stages of the infection,
2. The percentage of cells that need to receive the genetic construct in order to observe clinical effect,
3. The impact of one-off versus repeated delivery of gene therapy,
4. The impact of gene therapy in terms of inhibiting the emergence of ×4 viral strains.

## Methods

### Model equations

**Figure 1:**
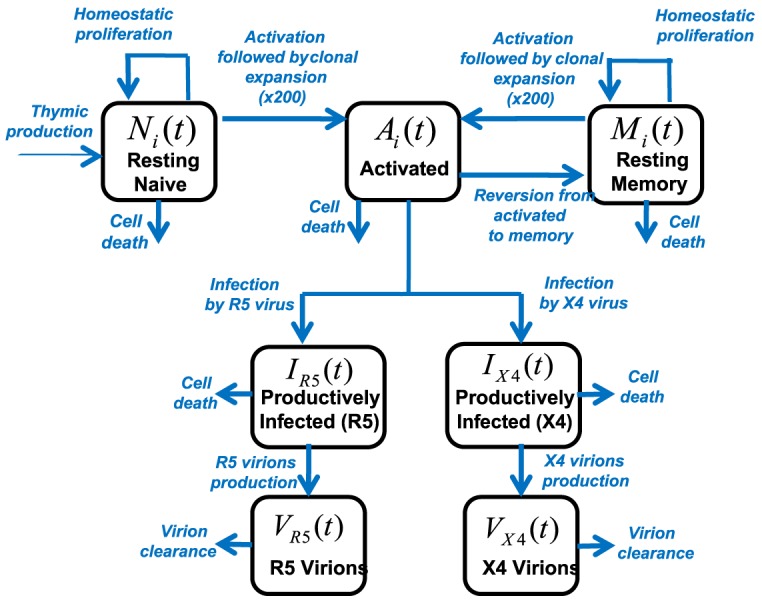
Compartment model of cellular and of viral dynamics. Here 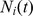, 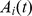 and 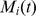 denote compartments of resting naive, activated and resting memory CD4+T cells respectively, with 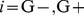 respectively denoting non gene-modified (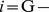) or gene-modified (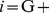) CD4+T cells. G+ CD4+T cells are assumed to contain a dual anti-HIV gene construct (CCR5 entry inhibitor + C46 fusion inhibitor, see Methods). The variables 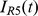 and 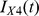 denote compartments of productively infected CD4+T cells with R5 and ×4 virus respectively. 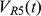 and 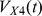 respectively denote compartments of R5 and ×4 virions.



The model employed here is depicted in Figure 1 and is described by the following differential equations, with all parameters listed in Table S1

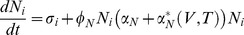


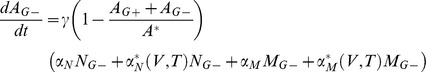


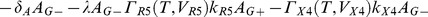





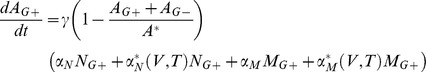





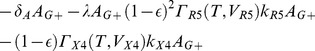





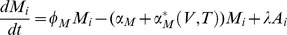





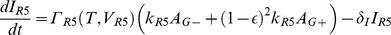





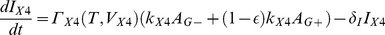





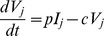
where the index 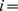 G-, G+ respectively denotes non gene-modified CD4+T cells (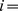 G-) and CD4+T cells containing the anti-HIV dual gene construct (CCR5 entry inhibitor + C46 fusion inhibitor, 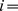 G+). Gene-modified (G+) CD4+T cells are assumed to be less susceptible to viral infection than non-gene–modified (G-) CD4+T cells (see below).

The variables 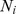, 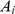 and 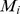 respectively denote the number of resting naive, activated and resting memory CD4+T cells at time 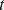 (in unit of days). The variables 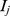 and 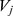 respectively denote the total number of productively infected cells of strain 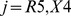 and the total number of viral particles of strain 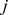.

The terms 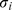 denote thymic export of naive CD4+T cells, with 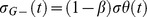 and 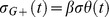 denoting the thymic export of G- and G+ CD4+T cells respectively. The term 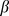 (here 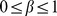) denotes the fraction of G+ CD34+ HSC in the bone marrow. Here 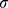 denotes total thymic output in a healthy individual. The term 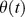 (here 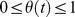) models reduction of thymopoiesis with duration of untreated infection 45]. It is assumed that 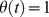 in a healthy individual. The term 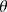 is governed by the equation 
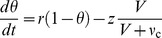
 (here 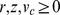 are parameters), where 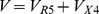 denotes the total number of viral particles. In our model, the presence of substantial viremia (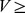 4 log10 HIV RNA copies/mL) reduces thymic supply (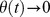), but lower levels of viremia (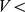 4 log10 HIV RNA copies/mL) results in restoration of thymic supply (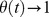).

The parameters 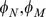 denote the net effect of homeostatic proliferation and of cell death in the compartment of resting *naive* and *memory* CD4+T cells respectively. 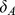 and 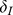 respectively denote death rates of activated and productively infected CD4+T cells.

The terms 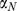 and 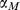 denote normal activation rates (in a healthy individual). The terms 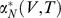 and 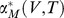 model HIV-induced activation of resting CD4+T cells [46], and are assumed to depend on the total viral load 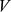 as well as the total number 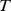 of CD4+T cells. Here 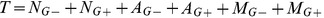. Since this total is mainly used in association with the number of target cells for infection and the pool of uninfected cells available for activation, we did not include the relatively small number of infected cells. These terms are defined by: 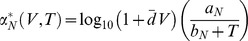





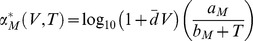
where 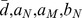 and 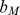 denote parameters, so that HIV-induced activation levels increase with higher total viral load 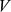 and with lower total CD4+T cell count 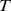.

The term 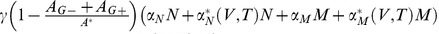 denotes clonal expansion following activation. Activated CD4+T cells expand by a factor of 
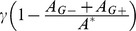
, resulting in approximately 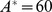 activated cells/ µL as observed during HIV infection [47]. The term 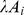 denotes reversion from the activated to the quiescent/resting state. Virions are produced at rate 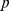 per day per infected cell and removed at rate 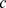.

Here 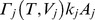 denotes the infection rate by viral strain 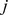 (here 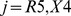) of target cells 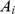 of phenotype 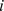 (here 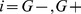), where 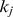 denotes the infectivity of viral strain 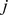 for target cells 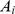. The terms 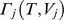 model increasing viral infection rates over the course of infection [48]–[50] and with accumulation of total viral load [51], resulting in higher viral loads with longer duration of infection:
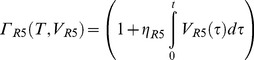





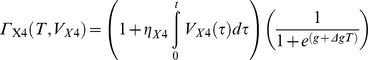



The term 
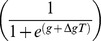
 models increased selection for ×4 virus with lower total CD4+T cell counts 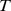 [40]–[42], reflecting increased availability of CXCR4-expressing activated target cells for productive infection by ×4 virus at lower total CD4+T cell counts [52], [53]. Here 
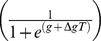
 increases monotonically with decreasing total CD4+T cell counts 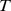, so that ×4 selection is driven by decreasing total CD4+T cell counts 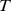. The parameters 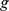 and 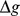 are selected so that ×4 emergence occurs with a median time of approximately 4 years post-infection. The time of ×4 emergence is defined in our model as the time at which the ×4 viral load exceeds a value of 100 HIV RNA copies/mL. The parameter 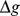 is drawn randomly from a uniform distribution (see Table S1), so that the 5^th^ and 95^th^ percentiles of ×4 emergence times are approximately 1 and 8 years respectively, thereby capturing the observed variability in the time of ×4 emergence [40]–[42], [54]. The simulations with no ×4 virus are produced by setting 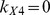.

### Model simulations and initial values for model variables

The effective viral and cell population sizes in our simulations are taken as the total numbers of virions/cells in the body. When scaling to numbers and concentrations in peripheral blood (PB), we assume a 5L PB volume and also that 5.5% of total CD4+T cell reside in PB [55]. The value of 5.5% was obtained from analysis of peripheral blood data on CD4 and CD8+T lymphocyte concentrations after aphereses conducted during a previous gene therapy trial in humans. The number of virions per ml of PB is then estimated as 5.5% of the total body load divided by the 5,000 mls of PB. Although simulations for CD4+T cells and HIV RNA copies are shown per µL and per mL of PB respectively (in the Results section), all calculations are determined over total numbers in the body.

The course of infection is simulated over a 10 year period. We assume that time 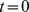 corresponds to the end of primary HIV infection (PHI), with a total CD4+T cell count in PB of 800 cells/ µL, such that initial levels of 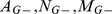 correspond to 60, 270 and 470 cells/ µL in PB respectively [56]

The number of R5 virions 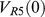 was initialized to a value of 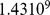 (corresponding to an R5 viral load of 4.5 log10 HIV RNA copies/mL in PB) and the initial number of productively infected cells 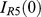 of R5 tropism was initialized to a value of 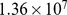 cells. The number of virions and number of productively infected cells for the ×4 virus were both initialized to zero.

The term 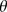 modelling decline of thymopoiesis is initialized to 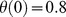, so that an individual at end of PHI (i.e. time 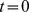) has a 20% reduction in thymopoesis compared to a healthy individual.

### Determination of model parameters

The model parameters are given in Table S1, and were selected from the literature, with unknown model parameters determined by model calibration against known in-vivo dynamics of CD4+T cells and viral loads.

The model was calibrated to capture the following dynamical aspects of the in-vivo biology:

1. Constant total CD4+T cell count of approximately 1000 cells/ µL in PB in a healthy individual, with 500 cells/ µL resting memory CD4+T cells and 450 cells/ µL resting naive CD4+T cells [57]. This contributed to the setting of naïve and memory cell proliferation rates.
2. Decline from approximately 800 cells/ µL PB at the end of PHI to below 200 cells/ µL at 8.4 years post-PHI for an individual infected with R5-tropic virus, in whom no ×4 tropic virus emerges [58]. R5 viral load of approximately 4.5 log10 HIV RNA copies/mL that increases to 5 log10 HIV RNA copies/mL after 10 years of untreated infection, reflecting increasing viral fitness (that is independent of viral tropism) with duration of infection [48], [49]. These factors determined feasible ranges for the infectivity rates and the HIV-induced activation rates.
3. Median time of ×4 emergence of approximately 4 years post-PHI [42], resulting in AIDS (<200 cells/ µL) within approximately 2 years following ×4 emergence [40]. This contributed to calculations for terms within 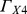.

### Reduction in viral infection rates for G+ CD4+T cells

In the present modelling G+ CD4+T cells contain a dual gene construct (CCR5 entry inhibitor + C46 fusion inhibitor). The CCR5 gene therapeutic inhibits infection by R5 virus as a result of CCR5 down-regulation on the cell surface [1], and we assume it reduces HIV infection by R5 virus by 92.5% (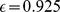
_)_ in line with in vitro analysis [59], but test sensitivity of results relative to an efficacy range of 87.5% to 97.5%. The C46 gene therapeutic inhibits fusion of both R5 and ×4 virus, and has been shown to reduce infection against R5 virus by 1 to 2 logs [23], [30], [60] and also against ×4 virus [30], [61]. In line with our assumptions for the CCR5 gene therapeutic and compatible with this viral load decrease we assume the C46 therapeutic also reduces HIV infection by 92.5% (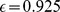
_)_. Hence with this combination gene therapy G+ CD4+T cells exhibit reduced likelihood of infection from R5 by an amount 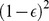 and from ×4 by an amount 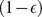.

### One-off and repeated delivery of gene therapy

For the case that the gene therapy is delivered as a one-off treatment to CD34+ HSC at time 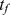 with 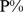 of CD34+ HSC receiving the gene construct, we set 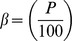 for times after 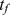, where 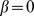 for 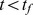. It is assumed that the percentage 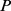 reflects the percentage of G+ CD34+ HSC in the bone marrow at steady state following engraftment. If the gene therapy is delivered repeatedly, then we assume that each repeated infusion results in 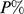 of the G- CD34+ HSC in the bone marrow receiving the gene construct. In particular, for any time-point at which therapy delivery is performed, if 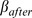 denotes the fraction (of G+ CD34+ HSC in the bone marrow) just after delivery and 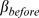 denotes the fraction just before delivery, then 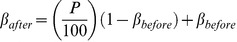.

Similarly for gene therapy delivered to CD4+T cells in PB with 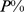 of CD4+T cells receiving the gene construct, we assume that 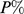 of the G- CD4+T cell subpopulations (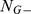,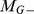,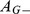) receive the gene construct. If the gene therapy is delivered repeatedly, then we assume that each repeated infusion results in 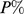 of the G- CD4+T cells receiving the gene construct.

### Reduced levels of bystander apoptosis in G+ CD4+T cells: Assumption +A1

The model assumptions from above represent the **standard scenario (STD)** considered in the present analysis. We also consider the impact of gene therapy subject to the following alternative assumption that acts to increase the impact of gene therapy in terms of preserving total CD4+T cell counts and decreasing viral loads:

#### Assumption +A1: Resting G+ CD4+T cells are less likely to undergo HIV-induced activation and subsequent bystander apoptosis

Under this assumption we replace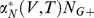 (which represents HIV-induced activation of resting naïve CD4+T cells) by 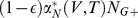 in the above model, and we also replace 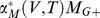 (which represents HIV-induced activation of resting memory CD4+T cells) by 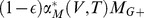. Here 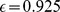 denotes the efficacy of the gene therapy from above.

Assumption +A1 (+A1) is motivated by previous reports that HIV *env* causes activation and subsequent bystander apoptosis through a CCR5 or CXCR4-dependent pathway in resting CD4+T cells, and that the levels of bystander apoptosis correlate with CD4+T cell surface expression of CCR5/CXCR4 chemokine co-receptors [62]–[66]. Another recent study also reported reduced bystander apoptosis if the entry step of the HIV infection cycle is inhibited by entry/fusion inhibitors [11]. In that ex vivo study with cultures of human tonsil tissue, it was also reported that over 95% of HIV-induced cell death is attributable to bystander apoptosis resulting from viral entry into a cell without viral integration into the cellular genome. Consequently Assumption A1 confers a selective advantage on resting G+ CD4+T cells, since these either have reduced CCR5 expression or inhibit the viral entry/fusion step of the HIV infection cycle, and should therefore be less likely to undergo activation and subsequent bystander apoptosis.

### Sensitivity analysis

As well as simulating the model with the parameters given in Table S1, we also performed a sensitivity analysis using 5,000 parameter sets determined from Latin Hypercube Sampling sampled uniformly with a ±10% variation of the parameters 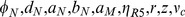, and a ±5% variation of 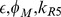. The sensitivity analysis was performed in the scenario when ×4 virus was not present. The parameter 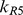
was limited to a 5% variation since its value was highly correlated with final CD4+T cell counts, while 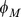
was highly correlated with final CD4+T cell counts under Assumption A1. In both cases a 5% variation limited variation around the mean of CD4+T cell counts to 25%.

All simulations were implemented in Mathworks MATLAB 2012a.

## Results

### Model calibration for course of untreated HIV infection

**Figure 2:**
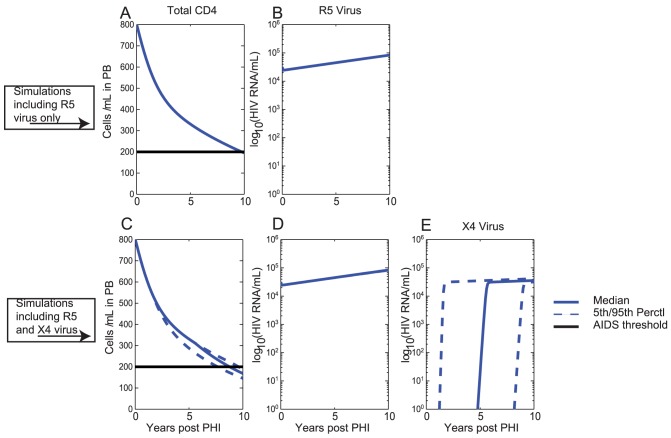
Progression of untreated HIV infection over a 10-year period. Here year 0 denotes the end of primary HIV infection (PHI). Solid lines denote median values, dashed lines denote the 5^th^ and 95^th^ percentile ranges. Also shown is the AIDS threshold of 200 cells/ µL (solid horizontal black line); A) Total CD4+T cell count and B) R5 Viral Load for a single simulation of untreated infection with R5 virus only (i.e. assuming 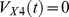 throughout the course of infection); C) Total CD4+T cell count, D) R5 Viral Load and E) ×4 Viral Load for Monte Carlo simulations (100 trials) of untreated infection assuming initial infection with R5 virus, but now including ×4 virus.

To calibrate the model, we first simulated the course ofuntreated HIV infection where an individual is infected with R5-tropic virus and in whom no ×4 virus is assumed (Figure 2 A,B). Here the total CD4+T cell counts decline from a value of 800 cells/ µL to a value below 200 cells/ µL (AIDS) after approximately 9 years (Figure 2 A) in line with the clinical course of untreated HIV infection with R5 tropic virus [58]. The total CD4+T cell count at the end of the simulated 11-year period (1 year post-infection plus an additional 10 years) is approximately 173 cells/ µL. Over the course of infection, the viral load exhibits an increase from a value of approximately 4.5 log HIV RNA copies/mL at year 0 to a value of 5 log HIV RNA copies/mL after 10 years (Figure 2 B), reflecting increasing viral fitness/diversity over the course of infection that is independent of viral tropism [48]–[50].

We also reproduce the course of untreated HIV infection where an individual is initially infected with R5-tropic virus but in whom ×4 virus can emerge (Figure 2 C,D,E), with selection for ×4 virus driven by decreasing total CD4+T cell counts in our model (see Methods). We performed Monte Carlo simulations with 100 trials, with the parameter 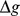 repeatedly sampled from a uniform distribution (see Methods and Table S1). Here ×4 emergence occurs with a median time of approximately 4 years post-PHI (Figure 2 E), resulting in accelerated progression to AIDS with a median time of AIDS of approximately 7.5 years post-PHI (Figure 2 C). Our model also captures significant variation in the time that ×4 emergence is observed (Figure 2 E) in line with clinical observations. At the end of the simulated 11 year period, the median total CD4+T cell count was 146 cells/ µL, which is considerably lower than the value of 173 cells/ µL with R5 virus only (Figure 2 A).

### One-off administration of therapy to CD4+T cells at 1 year post-PHI with 20% of CD4+T cells receiving gene construct

**Figure 3:**
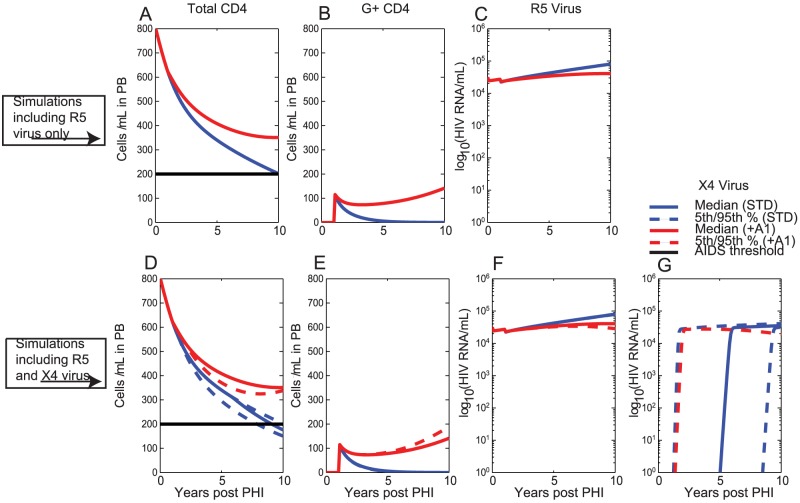
One-off delivery of gene therapy to CD4+T cells assuming that 20% of CD4+T cells receive the gene construct at year 1. Therapy is administered at year 1. Shown are the simulation outcomes under the standard scenario (STD) and under Assumption +A1 of reduced bystander apoptosis in G+ CD4+T cells (+A1); Solid lines denote median values, dashed lines denote the 5^th^ and 95^th^ percentile ranges for outcomes when ×4 virus can develop. Also shown is the AIDS threshold of 200 cells/ µL (solid horizontal black line); A) Total CD4+T cell count, B) G+ CD4+T cell count and C) R5 Viral Load for a single simulation with R5 virus only (i.e. assuming 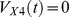 throughout the course of infection); D) Total CD4+T cell count, E) G+ CD4+T cell count F) R5 Viral Load and G) ×4 Viral Load for Monte Carlo simulations assuming initial infection with R5 virus, but now including ×4 virus. Monte Carlo simulations were performed for 100 trials and involved repeated sampling of parameter 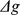 from a uniform distribution (see Methods and Table S1).

First we consider the case that gene therapy is delivered to CD4+T cells as a one-off treatment at 1 year post-PHI and with 20% of CD4+T cells receiving the gene construct (Figure 3). Simulation outcomes were determined with R5 virus only (Figure 3 A,B,C), and also when both R5 and ×4 viral strains were assumed in the simulations (Figure 3 D,E,F,G).

Under the standard scenario (STD), the total CD4+T cell counts and viral load are only marginally higher than for the untreated case from Figure 2, so that final median total CD4+T counts at the end of the 10 year period of 181 cells/ µL are observed if no ×4 virus is present will be the standard value for which we report (Figure 3 A,D, Table 1). Simulations with ×4 virus result in faster loss of CD4+T cells for an untreated individual and also less reconstitution of T cell counts especially with low levels of gene therapy (Table 2). The population of G+ CD4+T does not persist (Figure 3 B,E) due to their replacement with G- CD4+T cells from the thymus, resulting in negligible numbers of G+ CD4+T cells by 4 years post-PHI. The initial viral load decrease observed when therapy is delivered is not sustained for long (Figure 3 C,F).

**Table 1:** Total CD4+T cell counts in PB (cells/ µL) 10 years after commencement of therapy assuming no ×4 virus in these simulations when therapy is delivered to CD4+T cells – medians (5%, 95%) from sensitivity analyses.

Scenarios	Standard scenario (STD)					Scenario of reduced bystander apoptosis in G+ CD4+T cells (Assumption +A1)						
Percentage G+ cells →	10%	20%	30%	40%	50%	10%	20%	30%		40%		50%
**One-off year 1**	177 (124, 227)	181 (128, 230)	184 (133, 233)	188 (137, 237)	192 (141, 241)	307 (244, 406)	355 (278, 472)	385 (300, 514)	407 (316, 546)		426 (330, 573)	
**One-off year 4**	121 (57, 178)	124 (60, 180)	126 (63, 182)	128 (66, 185)	131 (69, 187)	325 (249, 438)	363 (280, 485)	385 (297, 515)	401 (310, 536)		413 (319, 554)	
**One-off year 7**	78 (0, 147)	80 (2, 145)	82 (3, 146)	84 (0, 147)	86 (1, 148)	385 (291, 507)	407 (315, 534)	419 (326, 551)	428 (333, 565)		435 (339, 575)	
**Years 1, 3, 5, 7, 9**	188 (137, 236)	202 (153, 250)	217 (169, 265)	233 (186, 281)	249 (203, 297)	389 (314, 501)	461 (372, 596)	511 (412, 661)	550 (444, 712)		582 (472, 752)	
**Years 4, 6, 8, 10, 12**	130 (72, 186)	142 (86, 197)	154 (101, 208)	168 (116, 220)	182 (131, 234)	389 (311, 504)	445 (358, 577)	484 (389, 629)	516 (414, 669)		543 (437, 704)	
**Years 7, 9, 11, 13, 15**	87 (68, 147)	98 (76, 156)	108 (81, 165)	120 (86, 175)	132 (92, 186)	418 (332, 540)	455 (364, 589)	483 (387, 625)	506 (405, 657)		526 (422, 684)	
**years 1, …, 10**	200 (150, 248)	226 (179, 274)	251 (205, 300)	276 (231, 325)	301 (256, 350)	450 (365, 577)	534 (434, 686)	590 (482, 755)	632 (519, 805)		665 (550, 842)	
**Years 4, …, 13**	140 (84, 195)	162 (110, 216)	185 (134, 237)	207 (158, 259)	230 (182, 281)	437 (353, 563)	505 (408, 650)	551 (447, 709)	588 (479, 753)		618 (507, 787)	
**Years 7, …, 16**	96 (76, 155)	115 (85, 171)	134 (95, 189)	154 (109, 207)	174 (128, 227)	449 (360, 577)	497 (401, 641)	533 (430, 688)	563 (455, 724)		589 (479, 754)	

Median final total CD4+T cell counts in PB (cells/ µL) 10 years after commencement of therapy when therapy is delivered to CD4+T cells, assuming no ×4 virus in these simulations (i.e. 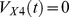 for all times 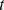). Also shown are the 5^th^ and 95^th^ percentile values of final CD4+T cell counts from the parameter sensitivity analysis. Simulation outcomes are shown for the standard scenario (STD) and under Assumption +A1 of reduced bystander apoptosis in G+ CD4+T cells. Outcomes are also shown for the case that therapy is delivered as one-off treatment, every 2 years or every 1 year. Cases that 10%, 20%, 30%, 40% and 50% of cells receive the gene construct are also simulated. First time of therapy administration was either 1 year, 4 years or 7 years post-PHI.

**Table 2:** Total CD4+T cell counts in PB (cells/ µL) 10 years after commencement of therapy when therapy is delivered to CD4+T cells, and ×4 virus can develop.

Scenarios	Standard scenario (STD)					Scenario of reduced bystander apoptosis in G+ CD4+T cells (Assumption +A1)				
Percentage G+ cells →	10%	20%	30%	40%	50%	10%	20%	30%	40%	50%
**One-off year 1**	149	152	155	159	163	305	355	383	403	419
**One-off year 4**	95	97	99	101	103	345	376	393	407	418
**One-off year 7**	57	58	60	61	63	413	429	437	443	446
**Years 1, 3, 5, 7, 9**	158	172	186	201	216	386	454	500	537	582
**Years 4, 6, 8, 10, 12**	103	113	123	135	147	398	446	481	510	534
**Years 7, 9, 11, 13, 15**	74	80	88	96	106	436	464	486	505	523
**years 1, …, 10**	169	194	218	243	271	443	522	582	632	665
**Years 4, …, 13**	111	130	150	172	194	438	499	542	579	613
**Years 7, …, 16**	80	93	108	123	140	460	498	529	556	583

Mean total CD4+T cell counts in PB (cells/ µL) 10 years after commencement of therapy when therapy is delivered to CD4+T cells, with ×4 virus included in the simulations. Monte Carlo sampling with 100 trials (repeated sampling of parameter 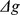 from a uniform distribution, see Methods) was performed for each case shown. The same scenarios as in Table 1 were simulated.

Substantially improved outcomes are achieved under the assumption of decreased bystander apoptosis of G+ CD4+T cells (+A1). Here the G+ CD4+T cells persist at stable levels due to their relative advantage in terms of lower activation even against new G- CD4+T cells exported from the thymus (Figure 3 B,E). This scenario results in substantial preservation of CD4+T cell counts, with median total CD4+T cell counts of 355 cells/ µL after 10 years (Figure 3 A,D, Tables 1, 2). A marked and sustained reduction in R5 viral load (Figure 3 C,F), as well as strong suppression of ×4 emergence (Figure 3 G), are also achieved.

### One-off administration of therapy to CD34+ HSC at 1 year post-PHI with 20% of CD34+ HSC receiving the gene construct

**Figure 4:**
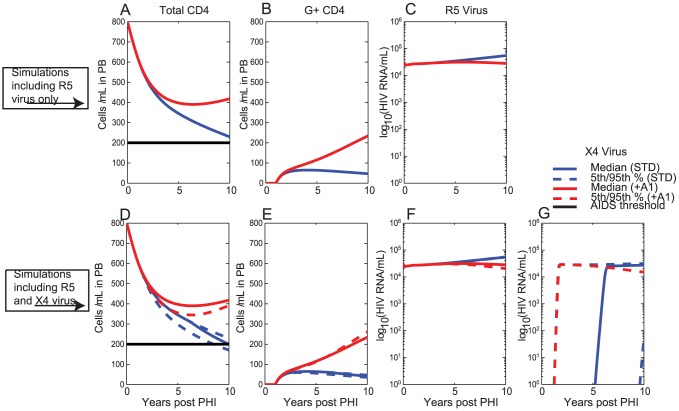
One-off delivery of gene therapy to CD34+ HSC assuming that 20% of CD34+ HSC in the bone marrow receive the gene construct at year 1. The legend for this figure is the same as for Figure 3.

Next we consider the impact when therapy is delivered to CD34+ HSC at 1 year post-PHI and as a one-off treatment with 20% of CD34+ HSC receiving the gene construct (Figure 4). Simulation outcomes were determined with R5 virus only (Figure 4 A,B,C, Table 3), and also when both R5 and ×4 viral strains were assumed in the simulations (Figure 4 D,E,F,G, Table 4).

**Table 3:** Total CD4+T cell counts in PB (cells/ µL) 10 years after commencement of therapy assuming no ×4 virus in these simulations when therapy is delivered to CD34+ HSC cells – medians (5%, 95%) from sensitivity analyses.

Scenarios	Standard scenario (STD)					Scenario of reduced bystander apoptosis in G+ CD4+T cells (Assumption +A1)				
Percentage G+ cells →	10%	20%	30%	40%	50%	10%	20%	30%	40%	50%
**One-off year 1**	191 (139, 240)	211 (161, 258)	235 (186, 280)	261 (215, 304)	292 (249, 333)	354 (303, 423)	432 (370, 515)	491 (423, 585)	545 (471, 644)	595 (518, 699)
**One-off year 4**	134 (72, 190)	152 (92, 207)	174 (114, 227)	199 (142, 250)	229 (174, 277)	371 (308, 462)	435 (364, 536)	485 (407, 593)	530 (448, 645)	575 (489, 694)
**One-off year 7**	89 (0, 154)	104 (66, 165)	122 (73, 181)	143 (83, 201)	170 (107, 226)	405 (329, 513)	449 (370, 562)	485 (402, 605)	520 (433, 644)	557 (467, 685)
**Years 1, 3, 5, 7, 9**	225 (175, 272)	287 (241, 329)	353 (312, 390)	417 (380, 448)	473 (441, 500)	460 (401, 537)	580 (511, 670)	666 (592, 762)	730 (655, 830)	777 (701, 879)
**Years 4, 6, 8, 10, 12**	164 (101, 219)	221 (163, 272)	288 (234, 334)	357 (309, 397)	422 (380, 457)	452 (386, 544)	558 (481, 661)	639 (557, 748)	704 (620, 816)	753 (669, 867)
**Years 7, 9, 11, 13, 15**	113 (60, 174)	162 (90, 221)	225 (158, 280)	296 (234, 345)	366 (310, 409)	460 (387, 566)	545 (463, 659)	616 (530, 735)	677 (588, 798)	726 (637, 848)
**years 1, …, 10**	271 (223, 314)	375 (335, 410)	460 (427, 488)	518 (489, 542)	554 (526, 576)	551 (485, 636)	687 (613, 782)	764 (689, 863)	807 (731, 908)	831 (754, 935)
**Years 4, …, 13**	205 (145, 257)	311 (259, 355)	407 (362, 443)	477 (438, 508)	522 (485, 551)	530 (457, 629)	658 (577, 765)	738 (655, 848)	785 (701, 898)	813 (728, 928)
**Years 7, …, 16**	148 (70, 208)	248 (181, 302)	350 (291, 395)	429 (376, 468)	482 (433, 519)	521 (443, 630)	634 (548, 751)	711 (622, 829)	759 (670, 879)	789 (699, 910)

Final total CD4+T cell counts in PB (cells/ µL) 10 years after commencement of therapy when therapy is delivered to CD34+ HSC cells, assuming no ×4 virus in these simulations (i.e. 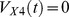 for all times 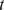). Descriptions as for Table 1.

**Table 4:** Total CD4+T cell counts in PB (cells/ µL) 10 years after commencement of therapy when therapy is delivered to CD34+ HSC, and ×4 virus can develop.

Scenarios	Standard scenario (STD)					Scenario of reduced bystander apoptosis in G+ CD4+T cells (Assumption +A1)				
Percentage G+ cells →	10%	20%	30%	40%	50%	10%	20%	30%	40%	50%
**One-off year 1**	161	180	202	227	259	352	425	481	531	580
**One-off year 4**	105	120	138	161	191	383	436	481	523	564
**One-off year 7**	69	80	94	110	132	425	460	490	520	552
**Years 1, 3, 5, 7, 9**	193	248	324	386	440	451	564	653	721	770
**Years 4, 6, 8, 10, 12**	129	182	243	306	376	453	548	629	694	745
**Years 7, 9, 11, 13, 15**	86	125	176	242	316	470	543	611	670	719
**years 1, …, 10**	233	346	428	485	520	537	675	755	800	825
**Years 4, …, 13**	164	264	358	435	482	523	648	728	776	805
**Years 7, …, 16**	114	196	295	381	436	522	627	703	750	779

Mean total CD4+T cell counts in PB (cells/ µL) 10 years after commencement of therapy when therapy is delivered to CD34+ HSC cells, with ×4 virus included in the simulations. Descriptions as for Table 2.

When gene therapy is delivered to CD34+ HSC under the standard scenario (STD), median total CD4+T cell counts of 211 cells/ µL are observed if ×4 virus is not present and 180 cells/ µL if it is (Figure 4 A,D, Tables 3, 4), both of which are higher than the corresponding values when therapy was delivered to CD4+T cells under the standard scenario STD. Furthermore G+ CD4+T cells persist at relatively constant levels (Figure 4 B,E) and do not decay as observed in the standard scenario when therapy was administered to CD4+T cells (Figure 3 B,E). A sustained viral load reduction is achieved in R5 virus (Figure 4 C,F).

Much higher impact was observed under the assumption of decreased bystander apoptosis of G+ CD4+T cells (A1), where median total median CD4+T cell counts of 432 cells/ µL were achieved at the end of 10 years (Figure 4 A,D).

In each of the two scenarios median total CD4+T cell counts at the end of the 10 year period were higher for one-off delivery to CD34+ HSC than for one-off delivery to CD4+T cells.

### Repeated annual administration of therapy to CD4+T cells from 1 year post-PHI with 20% of CD4+T cells receiving the gene construct

**Figure 5:**
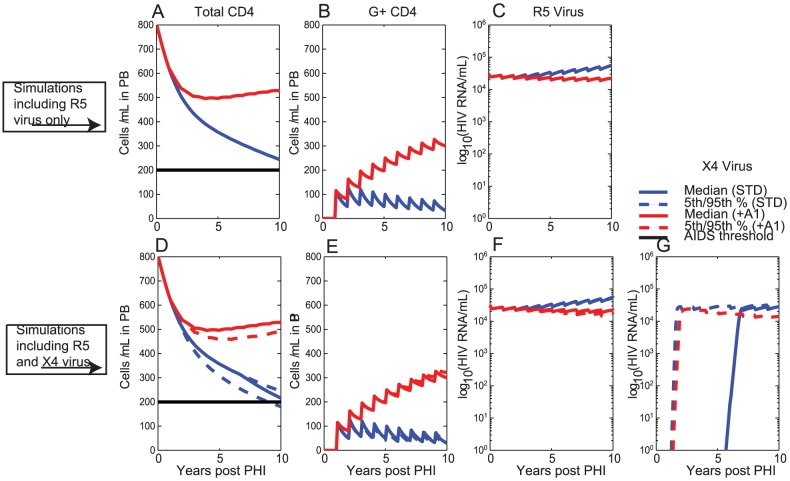
Repeated annual delivery of gene therapy to CD4+T cells assuming that 20% of CD4+T cells receive the gene construct every year from year 1 (i.e. every year, starting from year 1, 20% of G- CD4+T cells become G+ CD4+T cells). Therapy is first administered at year 1 and then annually thereafter. Shown are the simulation outcomes under the standard scenario (STD) and also under Assumption +A1 of reduced bystander apoptosis in G+ CD4+T cells (+A1); Solid lines denote median values, dashed lines denote 5^th^ and 95^th^ percentiles for outcomes when ×4 virus can develop. Also shown is the AIDS threshold of 200 cells/ µL (solid horizontal black line); A) Total CD4+T cell count, B) G+ CD4+T cell count and C) R5 Viral Load for a single simulation with R5 virus only (i.e. assuming 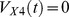 throughout the course of infection); D) Total CD4+T cell count, E) G+ CD4+T cell count F) R5 Viral Load and G) ×4 Viral Load for Monte Carlo simulations assuming initial infection with R5 virus, but now including ×4 virus.

We also considered the scenario that gene therapy is delivered to CD4+T cells repeatedly every year starting at 1 year post-PHI (Figure 5). Here at each time of therapy delivery (i.e. every year starting from year 1), 20% of G- CD4+T cells become G+ CD4+T cells. Simulation outcomes were determined with R5 virus only (Figure 5 A,B,C), and also when both R5 and ×4 viral strains were assumed in the simulations (Figure 5 D,E,F,G).

Under the standard scenario (STD), the impact is more pronounced with repeated delivery of therapy to CD4+T cells than with one-off delivery, so that median total CD4+T cell counts of 226 cells/ µL are observed after 10 years (Figure 5 A D). This is in contrast to one-off delivery of therapy to CD4+T cells under the standard scenario STD, where final total CD4+T cell counts of 181 cells/ µL were observed.

Most impact was achieved under the scenario of reduced bystander apoptosis of G+ CD4+T cells (+A1), with median total CD4+T cell counts of 534 cells/ µL (Figure 5 A,D) and substantial viral load inhibition at the end of the 10 year period (Figure 5 C,F,G). Substantial inhibition of ×4 viral strains was also observed under this scenario (Figure 5 G). Repeated administration of therapy under this scenario (+A1) results in improved outcomes over one-off administration of 534 cells/ µL compared to 355 cells/ µL (Table 1).

### Repeated annual administration of therapy to CD34+ HSC from 1 year post-PHI with 20% of CD34+ HSC receiving the gene construct

**Figure 6:**
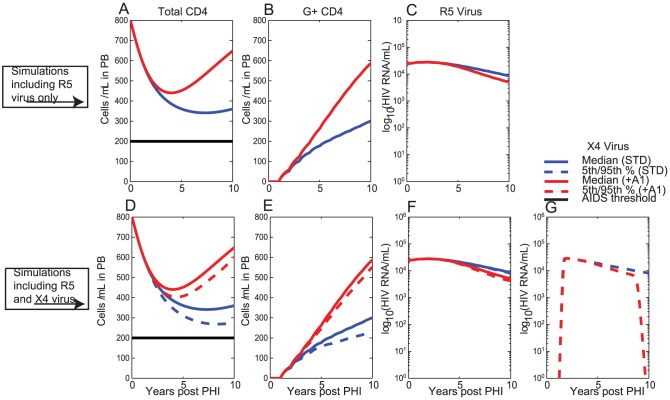
Repeated annual delivery of gene therapy to CD34+ HSC assuming that 20% of CD34+ HSC in the bone marrow receive the gene construct every year from year 1 (i.e. every year, starting from year 1, 20% of G- CD34+ HSC become G+ CD34+ HSC). The legend for this figure is the same as for Figure 5.

We also considered the scenario that gene therapy is delivered to CD34+ HSC every year starting at 1 year post-PHI (Figure 6). Here, at each time of therapy delivery (i.e. every year starting from year 1), 20% of G- CD34+ HSC become G+ CD34+ HSC. Simulation outcomes were determined with R5 virus only (Figure 6 A,B,C, Table 3), and also when both R5 and ×4 viral strains were assumed in the simulations (Figure 6 D,E,F,G, Table 4).

Substantial preservation of total CD4+T cell counts was observed (Figure 6 A,D), resulting in median total CD4+T cell counts of 375 cells/ µL at the end of 10 years for the STD scenario and 687 cells/ µL under scenario +A1, when ×4 virus did not emerge. The population of G+ CD4+T cells persists and even expands under each scenario (Figure 6 B,E), albeit at different rates for each of the two scenarios. Under the +A1 scenario, both R5 and ×4 viral load were driven well below 10,000 HIV RNA copies/mL within 10 years.

These final total CD4+T cell counts are higher than the corresponding values for the case that therapy is delivered repeatedly to CD4+T cells. Repeated delivery to CD34+ HSC also resulted in improved outcomes over one-off delivery to CD34+ HSC.

### Simulation outcomes under variations in percentage of cells receiving the gene construct, in timing of commencement of therapy and in frequency of therapy administration

To further explore the impact of gene therapy under the two scenarios of interest (STD, +A1), we also considered the long-term impact of gene therapy under the following cases:

- The percentage of cells receiving the gene construct is 10%, 20%, 30%, 40% or 50%,
- The case that therapy is first administered at early (i.e. 1 year post-PHI), intermediate (i.e. 4 years post-PHI) or late (i.e. 7 years post-PHI) stage of the infection,
- The case that therapy is administered as a one-off treatment, or that therapy is administered repeatedly either every 1 year or every 2 years

Outcomes were determined in terms of total CD4+T cell counts 10 years after commencement of therapy, i.e. if therapy was first delivered at time 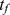, then outcomes were determined in terms of total CD4+T cell counts at 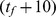 years. The final total CD4+T cell counts, for therapy delivery to CD4+T cells and to CD34+ HSC, are shown in Tables 1 and 3 respectively (simulations with R5 virus only) and in Tables 2 and 4 (simulations including both R5 and ×4 virus). The differences, in outcomes for final total CD4+T cell counts, between therapy delivery to CD34+ HSC versus therapy delivery to CD4+T cells are given in Tables 5 and 6.

**Table 5:** Differences between entries of Tables 3 and 1 (delivery to CD34 minus delivery to CD4), in the absence of ×4 virus.

Scenarios	Standard scenario (STD)					Scenario of reduced bystander apoptosis in G+ CD4+T cells (Assumption +A1)				
Percentage G+ cells →	10%	20%	30%	40%	50%	10%	20%	30%	40%	50%
**One-off year 1**	14	30	51	73	100	47	77	106	138	169
**One-off year 4**	13	28	48	71	98	46	72	100	129	162
**One-off year 7**	11	24	40	59	84	20	42	66	92	122
**Years 1, 3, 5, 7, 9**	37	85	136	184	224	71	119	155	180	195
**Years 4, 6, 8, 10, 12**	34	79	134	189	240	63	113	155	188	210
**Years 7, 9, 11, 13, 15**	26	64	117	176	234	42	90	133	171	200
**years 1, …, 10**	71	149	209	242	253	101	153	174	175	166
**Years 4, …, 13**	65	149	222	270	292	93	153	187	197	195

Differences in final total CD4+T cell counts (for each simulated case from Tables 1 and 2) between therapy delivery to CD4+T cells and therapy delivery to CD34+ HSC, assuming no ×4 virus in the simulations. Positive entries denote cases where therapy delivery to CD34+ HSC results in higher final total CD4+T cell counts than therapy delivery to CD4+T cells.

**Table 6:** Differences between entries of Tables 4 and 2, when ×4 virus can develop.

Scenarios	Standard scenario (STD)					Scenario of reduced bystander apoptosis in G+ CD4+T cells (Assumption +A1)				
Percentage G+ cells →	10%	20%	30%	40%	50%	10%	20%	30%	40%	50%
**One-off year 1**	13	28	46	68	96	47	71	99	128	160
**One-off year 4**	11	23	39	60	87	38	60	88	116	147
**One-off year 7**	13	22	34	49	68	13	31	53	77	106
**Years 1, 3, 5, 7, 9**	35	76	138	185	224	66	111	153	184	187
**Years 4, 6, 8, 10, 12**	26	69	120	172	229	55	102	148	185	210
**Years 7, 9, 11, 13, 15**	12	45	88	146	210	34	79	125	165	196
**years 1, …, 10**	63	151	210	242	249	94	153	173	168	160
**Years 4, …, 13**	53	135	209	263	288	85	149	186	197	191

Differences in final total CD4+T cell counts (for each simulated case from Tables 1 and 2) between therapy delivery to CD4+T cells and therapy delivery to CD34+ HSC, with ×4 virus included in the simulations. Positive entries denote cases where therapy delivery to CD34+ HSC results in higher final total CD4+T cell counts than therapy delivery to CD4+T cells.

Under both scenarios (STD and +A1), therapy delivery to CD34+ HSC resulted in better outcomes in terms of final total CD4+T cell counts over therapy delivery to CD4+T cells (Tables 5 and 6). Even though this was observed for both the standard scenario STD and for scenario +A1, the effect was substantially more pronounced for the standard scenario STD.

Our modelling determined that even with only 10% of CD34+ HSC in the bone marrow receiving the gene construct as a one-off treatment, final total CD4+T cell counts of >360 cells/ µL could be achieved provided Assumption +A1 held (Tables 3 and 4). This was observed for therapy commencement at any stage of the infection (i.e. at 1, 4 or 7 years post-PHI) as well as when ×4 virus was included. These results indicate that substantial increases in total CD4+T cell counts can be achieved, even if therapy is first administered at later stages of the infection and even if only a small percentage of total cells receive the gene construct.

We observed that one-off administration of therapy to CD4+T cells under the standard scenario STD generally resulted in limited clinical impact (with final total CD4+T cell counts of <200 cells/ µL; see Tables 1 and 2), and only slightly better outcomes under scenario STD could be achieved with repeated therapy administration to CD4+T cells. Repeated therapy administration to CD34+ HSC (therapy delivery every 1 or 2 years) generally achieved much better results than single delivery to CD34, in contrast to the muted improvement for delivery to CD4 (Tables 3 and 4). Outcomes with repeated therapy administration under Assumption +A1 resulted in even better outcomes.

We observed that, under the standard scenario STD, commencement of therapy at earlier stages of the infection always resulted in higher final total CD4+T cell counts than commencement of therapy at later stages of the infection (Tables 1, 2, 3, 4). In contrast, under Assumption +A1, we observed that commencement of therapy at later stages could in some instances result in better outcomes (i.e. higher final total CD4+T cell counts) than commencement at early stages. This effect under Assumption +A1 was generally observed when therapy was delivered as a one-off treatment. When this “non-linear” effect was observed viral load (and also the viral load accumulation with time) was higher when therapy was commenced late than when commenced early, and resulted in increased selection for G+ CD4+T cells. This observed effect was however only substantially pronounced under Assumption +A1.

We also observed that, under Assumption +A1, in some instances final total CD4+T cell counts were higher when both R5 and ×4 viral strains were included in the modelling than when R5 virus only was included (compare Tables 1 and 2, also Tables 3 and 4). This effect in our modelling was again attributable to the fact that higher viremia (due to ×4 emergence) resulted in increased selection for G+ CD4+T cells for the same reasons as outlined above. The effect was most pronounced when therapy was commenced at a later stage of the infection (i.e. at 4 or 7 years post-PHI) and/or when the percentage of cells receiving the gene construct was low.

In summary Assumption A1 delivers a marked increase of CD4+T cell counts regardless of the delay before commencement of therapy. Delivery to CD34+T cells remains superior to direct gene delivery to CD4+T cells in all cases.

## Discussion

In the present analysis we evaluated the long-term impact on the course of HIV infection when a dual anti-HIV gene construct (CCR5 entry inhibitor +C46 fusion inhibitor) is delivered to either CD4+T cells or to CD34+ HSC. Previous computational studies have established that gene constructs that inhibit the early stages of the HIV infection cycle (i.e. pre-integration stages including entry/fusion steps) are more likely to achieve better long-term outcomes than those that inhibit the later stages [10], [67]–[69]. In the present study we determined the impact of delivering entry/fusion inhibitors to either CD4+T cells or to CD34+ HSC, in terms of preservation of total CD4+T cell counts, as well as in terms of inhibition of both R5 and ×4 viral loads, over a 10 year period.

Our modelling determined that gene therapy delivery to CD34+ HSC resulted in better outcomes than delivery to CD4+T cells in all circumstances (Tables 5, 6). When therapy was delivered to CD34+ HSC, a gradual accumulation of a sizeable, but persistent, population of G+ CD4+T cells (Figure 4, 6) was observed, resulting in the gradual exertion of protective effects by the gene therapy. In contrast, therapy delivery to CD4+T cells resulted in an immediate population of G+ CD4+T cells, but limited persistence/expansion of G+ CD4+T cells under this scenario (Figure 3, 5). The gradual accumulation of protective effects, when therapy was delivered to CD34+ HSC, is in agreement with previous reports that CD4+T cell export due to thymopoesis is a slow process [2], [5] estimated to contribute approximately 1 CD4+T cell/ µL/day in PB [70]. Thymic export rates are likely to be even lower in HIV-infected individuals, since thymopoiesis declines with duration of HIV infection [45], [71], as modelled in the present analysis, but that can also partially reconstitute at least with cART [72].

In the present modelling we determined that gene therapy delivery to CD34+ HSC can achieve greater clinical impact than with delivery to CD4+T cells, so that even a one-off gene therapy delivery to 20% of CD34+ HSC in the bone marrow resulted in final total CD4+T cell counts of 211 cells/ µL after 10 years under the standard scenario STD for the case that R5 virus only was modelled. If the uninfected G+ CD4+T cells (in addition to exhibiting reduced likelihood of becoming productively infected) also exhibited reduced levels of bystander apoptosis over G- CD4+T cells (i.e. under Assumption +A1), then with 10% of CD34+ HSC in the bone marrow receiving the gene construct as a one-off treatment, final total CD4+T cell counts of 354 cells/ µL. In contrast however, in-vivo studies to-date have reported very low levels of gene-marking in PB post-infusion with little or no clinical impact. These previous in-vivo studies have employed a number of anti-HIV gene constructs, including an anti-HIV OZ1 ribozyme [25], [28], a rev-responsive element decoy gene [27], a humanized dominant-negative REV protein (huM10) [29] and a triple construct that included a CCR5 ribozyme [26]. This discrepancy between the present modelling and previous in-vivo studies is most likely attributable to low engraftment levels (following infusion and equilibration) of gene-modified CD34+ HSC in bone marrow in those previous studies [73], possibly since no ablative regimens (cytoreduction) were performed. Bone marrow ablation was performed in one study in HIV-infected individuals with leukaemia [26], but in that study only a low percentage (<0.2%) of total CD34+ HSC received the gene construct, which resulted in persistent albeit low-level gene marking in peripheral blood (<0.2%) at 18 months post-infusion. In contrast, studies in mice employing bone marrow pre-conditioning using irradiation have demonstrated substantial expansion of gene-protected CD4+T cells and substantial anti-viral effect of therapy delivered to CD34+ HSC when CCR5 inhibitors were used [32], [74]. Hence higher engraftment levels should result in greater impact of gene therapy delivered to CD34+ HSC, as indeed observed in the present analysis. Myeloablation in the context of this modelling of delivery of gene-containing HSC would only be feasible for one-off delivery.

A recent study of repeated infusions of autologous CD4+T cells containing a lentiviral vector expressing an anti-sense gene complementary to HIV *env*, determined no additional persistence of the gene-containing cells with multiple infusions [75], unlike our calculations where repeated infusions always produced substantially higher CD4+T cell counts after 10 years. One possible explanation for this discrepancy relates to the function of the gene therapy. We and others have postulated, with support from mathematical modelling, that only Class 1 gene therapies that inhibit infection rather than only suppressing viral replication post-infection, will be effective [9], [67]. Both gene therapies modelled here are Class 1 whereas the therapy in the above study was not. However it may be that multiple infusions will be less effective than described here with the majority of effect achieved with the first infusion and decreasing returns from subsequent therapies.

In-vivo delivery of gene therapy to CD4+T cells can possibly provide an immediate protective/anti-viral effect, with any subsequent persistence/expansion of G+ CD4+T cells only likely to be observed if the G+ CD4+T cells are subject to substantially increased in-vivo selection over G- CD4+T cells. Recent results describing zinc finger nuclease CCR5 gene modification of autologous CD4+T cells showed an immediate impact on CD4+T cell levels [24]. Although these gene-modified cells decreased over time they did so at significantly slower rates than non-gene-modified CD4+T cells demonstrating a strong protective effect of this gene therapy. In the absence of a strong survival advantage, any expansion of G+ CD4+T cells would be expected to occur as a result of cell division/proliferation, which is a slow process that has previously been estimated at approximately 1 division every 3.5 years for naive T cells and 1 division every 22 weeks for memory T cells [76]. Previous modelling has demonstrated that in the absence of a strong selective advantage and sole reliance on cell division for expansion, G+ CD4+T cells are out-diluted and replaced by the thymic supply of G- CD4+T cells [68]. This is also in line with reports from clinical trials to-date, where gene-marking in PB was generally observed to decay with a half-life in the span of months following infusion [17]–[23], with recent studies reporting gene-marking detection at 10 years post-infusion but at extremely low levels [22] (0.01% to 0.1% of PBMCs expressing the gene construct). In our modelling we observed that if G+ CD4+T cells only exhibited reduced likelihood of productive infection (i.e. under standard scenario STD), then limited persistence/expansion of G+ CD4+T cells and little therapeutic impact was achieved with one-off delivery of therapy to CD4+T cells (Figure 3; Tables 1, 2). In contrast, if G+ CD4+T cells furthermore also exhibited reduced levels of bystander apoptosis (i.e. under Assumption +A1), then long-term persistence/expansion of G+ CD4+T cells and substantial preservation of total CD4+T cell counts were observed even with one-off therapy delivery to CD4+T cells. These results therefore indicate that any reduced levels of bystander apoptosis in G+ CD4+T cells can confer a strong selective advantage on G+ CD4+T cells, resulting in long-term persistence/expansion of G+ CD4+T cells and substantial preservation of total CD4+T cell counts. Inhibition of bystander apoptosis by these gene therapies is based on their ability to restrict HIV *env* binding and subsequent fusion with the cell membrane [11]. The ability of these gene constructs to achieve this additional aspect is supported by recent reports that C46 delivered to HSC of pigtail macaques provided positive selection of gene containing cells in peripheral blood and tissue, as well as enhanced CTL function and antibody responses [77].

That anti-HIV gene constructs containing a CCR5 entry inhibitor and a C46 fusion inhibitor can result in reduced levels of bystander activation and apoptosis *in-vivo* (as modelled by Assumption +A1 in the present analysis) is supported by a number of previous studies. It has been reported that levels of bystander apoptosis correlate with the surface expression of CCR5/CXCR4 [62]–[66]. It has also been reported that levels of bystander apoptosis correlate with the fusogenic activity of *env* [66], while recent results characterize high levels of depletion of non-productively infected cells through caspase-1-mediated pyroptosis [78], [79]. Consequently these previous studies predict a strong survival advantage for G+ CD4+T cells containing the dual construct (CCR5 entry inhibitor + C46 fusion inhibitor), since these have reduced CCR5 expression (due to CCR5 entry inhibitor) and inhibit the viral fusion step of the HIV infection cycle (due to C46 fusion inhibitor), and should therefore be less likely to undergo bystander apoptosis. Previous in situ labelling studies of lymph nodes from HIV-infected children and SIV-infected macaques have also reported that CD4+T cell depletion occurs predominately as a result of bystander apoptosis rather than as a result of productive infection of a cell [63], with over 95% of HIV-induced cell death attributable to bystander apoptosis resulting from viral entry into a cell prior to viral integration into the cellular genome [11]. Collectively therefore, entry/fusion inhibitors can result in reduced levels of bystander apoptosis from these processes and may achieve substantial in-vivo preservation of total CD4+T cell counts, as indeed observed in the present computational analysis. These therapies however would not ameliorate any increased activation and death associated with the heightened cytokine milieu, which would reduce the impact of Assumption A1.

Most impact with delivery of gene therapy is likely to be achieved in viremic patients, as opposed to patients with controlled/undetectable viral loads on stable cART. Previous in-vivo studies have reported increased selection for G+ CD4+T cells during standard treatment interruptions or in patients with substantial/detectable viral loads. This was observed for a number of anti-HIV genetic constructs in previous studies [20], [26], [29], [34]–[36]. Increased selection for G+ CD4+T cells under viremic conditions has also been reported in-vitro and in mouse studies employing CCR5 inhibitors [32], [33], [74]. While these studies have provided an indication of increased selection for G+ CD4+T cells due to viremia, the results of the present modelling now indicate that such effects are also likely to be observed in-vivo in the long-term. While in our modelling the reduced likelihood of productive infection in G+ CD4+T cells conferred a selective advantage over G- CD4+T cells in the presence of viremia, we observed strongest selection for G+ CD4+T cells if these cells furthermore also exhibited reduced levels of bystander apoptosis compared to G- CD4+T cells (i.e. under Assumption +A1). Collectively therefore these results indicate that the presence of viremia is likely to result in higher levels of cell death (following productive infection of the cell) and/or bystander apoptosis in G- CD4+T cells, resulting in the preferential depletion of this “unprotected" G- CD4+T cell subset and thereby driving the preferential expansion of the subset of G+ CD4+T cells.

A significant concern with gene constructs employing CCR5 inhibitors relates to the possibility of increased selection for ×4 viral strains, which are associated with accelerated progression to AIDS [40v42,52,53,80]. This concern is motivated by previous reports of increased ×4 tropism following administration of the CCR5 inhibitor CMPD 167 in three macaques [39]. The recent MOTIVATE clinical trials in HIV infected individuals also reported increased ×4 tropism following administration of the CCR5 inhibitor maraviroc [37], [38]. This particular aspect of increased ×4 selection when the CCR5 co-receptor is inhibited/down-regulated was not modelled explicitly in the present analysis (X4 selection in our model was driven by decreasing total CD4+T cell counts and *not* as a result of direct therapy pressure, see Methods). There are two reasons as to why this should not represent a substantial shortcoming of the present modelling. Firstly in the present modelling a dual construct (CCR5 entry inhibitor + C46 fusion inhibitor) was employed. Therefore, despite CCR5-downregulation in G+ CD4+T cells, ×4 selection is likely to be mitigated by the C46 fusion inhibitor that acts to inhibit ×4 viral entry into these G+ CD4+T cells. Secondly, strong selection for ×4 is only likely to be observed if the G+ CD4+T cells (containing the CCR5 inhibitor construct) constitute the majority of total CD4+T cells. The presence of a subpopulation of G- CD4+T cells at all times (as in the present modelling) is likely to sustain sufficient wild-type (and R5 tropic) viral replication in the population of G- CD4+T cells, thereby mitigating selection for ×4 virus [67], [68]. This bipolar partition into G+ and G- CD4+ T cells under gene therapy is in stark contrast to the scenario under traditional antiretroviral drugs (including the CCR5 inhibitor maraviroc) that bathe all cells in some inhibitory concentration of the drug thereby resulting in increased likelihood of selection for resistant mutants [67], [68]. Nevertheless the increasing likelihood of ×4 and dual-tropic virus with lower CD4+T cell count may add support for delivery of this combination gene therapeutic to early stages of infection.

Potential shortcomings of the present modelling relate to the additional effect of gene therapy on cell populations other than CD4+T cells, given that G+ CD34+ HSC also differentiate into macrophages and monocytes that are susceptible to HIV infection [3], [5]. Since this was not modelled in the present analysis, it is therefore likely that the present modelling outcomes represent an underestimate of the true benefit of gene therapy delivery to CD34+ HSC, as the establishment of a population of G+ macrophages/monocytes would result in additional protective benefits from therapy delivery to CD34+ HSC. Our modelling also did not include ×4 infection of CD34+ HSC. Previous studies reported that ×4 viral strains can infect CD34+ HSC [81], so that delivery of a protective gene construct (containing a C46 inhibitor that inhibits ×4 infection) to CD34+ HSC is likely to confer an additional survival advantage on G+ CD34+ HSC. However given the lack of quantitative data on HIV infection of HSC, this aspect was not modelled in the present analysis.

Finally, we did not model the emergence of viral strains that exhibit resistance to the present dual construct (however we did model ×4 emergence but this was as a result of lower total CD4+T cell counts and not as a result of direct therapy pressure, see previous paragraph). This should however not significantly impact on our conclusion, given that previous studies reported that the presence of a significant population of G- CD4+T cells at all times (as in our modelling) ensures sufficient wild-type virus replication, so as to mitigate the emergence of viral strains resistant to the gene therapy [67], [68]. Our previous modelling determined that 4 independent short-hairpin RNA (shRNA) anti-HIV constructs (acting independently and each with an 80% efficacy) are required to mitigate the emergence of viral mutants resistant to the gene therapeutic [67]. This implies that a 99.84% overall efficacy by the 4 shRNA constructs (here 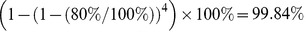) mitigates viral resistance. The dual construct employed in the present analysis however assumed a 92.5% mean efficacy of each construct, giving a 99.44% overall efficacy against R5 tropic virus assuming the two constructs act independently (here 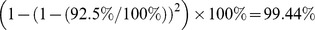)). This figure for likely overall efficacy of the dual construct is comparable to the overall efficacy estimated previously with 4 shRNA, so that resistance to the present dual construct is likely to be mitigated sufficiently. Further to the point, the dual construct employed in our modelling inhibits a cellular process that is less susceptible to mutation than the viral processes targeted by the shRNA [67]. Hence the present dual vector will be a superior therapeutic to the 4 shRNA therapy that suppressed resistance in our previous modelling.

In conclusion we have demonstrated that gene therapy employing entry/fusion inhibitors can achieve substantial clinical impact in terms of long-term preservation of total CD4+T cells counts and forestalment of AIDS. Importantly, this was observed even if only a subset of total cells received the gene construct, indicating that full immune system ablation is not necessary (prior to delivery of the gene therapy) in order to achieve substantial clinical impact. We determined that therapy delivery to CD34+ HSC generally resulted in better outcomes than therapy delivery to CD4+T cells. Maximal impact in our modelling was observed if the uninfected G+ CD4+T cells, in addition to having reduced likelihood of productive infection, exhibited lower levels of bystander apoptosis over G- CD4+T cells. Under this scenario therapy delivery to either CD4+T cells or to CD34+ HSC resulted in substantial preservation of total CD4+T cell counts. The present mathematical modelling demonstrates that gene therapy employing entry/fusion inhibitors represents a promising and potent anti-HIV modality, and that further clinical investigation of these gene therapeutics is more than justified.

## Supporting Information

Table S1 **Model parameters and values.**
(PDF)
