# Supplementary material for: A Quantitative Comparison of Anti-HIV Gene Therapy Delivered to Hematopoietic Stem Cells versus CD4+ T Cells
Source: PLoS Comput Biol. 2014 Jun 19;10(6):e1003681. doi: 10.1371/journal.pcbi.1003681 (PMC4063676; doi:10.1371/journal.pcbi.1003681)
Supplement: Table S1 — Model parameters and values. (PDF) [file pcbi.1003681.s001.pdf]

Supplementary Table S1: Model parameters and values

Parameter	Parameter description	Parameter value	Source
σ	Total thymic export rate in healthy individual	$\sigma = 1.818 \times 10^8$ cells/day	From [1]. Results in supply of 2 cells/µL/day in PB. See also [2].
δ_R	Death rate of resting cells	2.8×10^{-4} /day	From [3]. See also [2].
π_N	cell proliferation and cell death in compartment of resting *naïve* CD4+ T cells	7.08×10^{-4} /day	From [3]. See also [2].
π_M	cell proliferation and cell death in compartment of resting *memory* CD4+ T cells	0.0065 /day	From [3]. See also [2].
α_N<br>α_M	Normal activation rates of resting naive (α_N) and resting memory cells (α_M)	$\alpha_N = 0.0050$ /day<br>$\alpha_M = 0.0072$ /day	Determined by calibration to give constant total CD4+ T cell levels of approximately 1000 cells/µL in PB in a healthy individual, with 500 cells/µL memory and 450 cells/µL naive CD4+ T cells [4].
γ	Parameter determining clonal expansion following cell activation	$\gamma = 200$	From [5,6]. Assumes that a cell undergoes 5 to 10 mitiotic cycles during division following activation. The choice $\gamma = 200$ assumes $\log_2 200 \approx 8$ mitiotic cycles following activation.
A^*	Setpoint determining number of activated CD4+ T cells	$A^* = 5.454 \times 10^9$ cells	From [7]. Results in approximately 60 cells/µL activated CD4+ T cells in PB throughout course of HIV infection.
δ_A	Death rate of activated cells	$\delta_A = 1/6$ /day	From [5]. Implies half-life of approximately 5 days for activated CD4+ T cell.
λ	Deactivation rate of activated cells, resulting in new memory cells	$\lambda = 0.0083$ /day	From [5,7]. Here $\lambda = 0.05\ \delta_A$. Assumes that 95% of activated CD4+ T cells die, with 5% reverting to memory.

θ	Parameter modelling reduction of thymopoiesis with duration of infection [8] . Decline assumed to occur as a result of presence of viral load. Here θ is governed by the differential equation : $\dot{\theta} = r(1-\theta) - z\,\frac{\mathrm{V}}{\mathrm{V}+\mathrm{v_c}}$.	$r = 5.48 \times 10^{-4}$<br>$z = 6.84 \times 10^{-4}$<br>$v_c = 9.09 \times 10^{8}$ virions	Calibrated to result in AIDS (<200 cells/µL in PB) at 8.9 years post-PHI with R5 virus only. Also gives naive CD4+ T cells increases of approximately 100 cells/µL in PB over 2 years on cART with 90% efficacy [9] when cART is commenced at 4 years post-PHI.
ϕ_N	Net effect of cell proliferation and cell death in compartment of resting *naïve* CD4+ T cells	$\phi_N = (\pi_N - \delta_N)$, where $\pi_N = 7.08 \times 10^{-4}$ /day and $\delta_N = 2.8 \times 10^{-4}$ /day	Here π_N (proliferation rate of naive cells) and δ_N (death rate of naive cells) are from [3].
ϕ_M	Net effect of cell proliferation and cell death in compartment of resting *memory* CD4+ T cells	$\phi_M = (\pi_M - \delta_M)$, where $\pi_M = 0.0065$ /day and<br>$\delta_M = 2.8 \times 10^{-4}$ /day	Here π_M (proliferation rate of memory cells) and δ_M (death rate of memory cells) are from [3].
$\alpha_N^*(V,T)$<br>$\alpha_M^*(V,T)$	Terms modelling HIV-induced activation and subsequent bystander apoptosis. Here :<br>$\alpha_N^*(V,T) = \log_{10}(1+\bar{d}V)\left(\frac{a_N}{b_N+T}\right)$<br>$\alpha_M^*(V,T) = \log_{10}(1+\bar{d}V)\left(\frac{a_M}{b_M+T}\right)$	$\bar{d} = 2.2 \times 10^{-5}$ /virions<br>$a_N = 1.19 \times 10^{8}$ /cells/day<br>$b_N = 3.99 \times 10^{11}$ cells<br>$a_M = 2.24 \times 10^{7}$/cells/day<br>$b_M = -5.86 \times 10^{8}$ cells	Determined to give decline of CD4+ T cells during untreated infection with R5 virus only, so that AIDS occurs approximately 8 years post-PHI. These terms ensure increase activation with higher viral loads and also with lower total CD4+ T cell counts. The terms $\alpha_N^*(V,T)$, $\alpha_M^*(V,T)$ are always positive in our simulations.

k_{R5}<br>k_{X4}	Rates of infectivity of R5 virus and of X4 virus for CD4+ T cells, in the absence of gene therapy.	$k_{R5} = 1.25 \times 10^{-9}$<br>$k_{X4} = 1.63 \times 10^{-9}$<br>where each therapy has mean efficacy of $\epsilon = 0.925$	Determined to give R5 viral load that increases from 4.5 to 5 log10 HIV RNA copies/mL in PB during untreated infection. Determined to give accelerated progression to AIDS after X4 virus emerges. The choice of $\epsilon = 0.925$ assumes 92.5% efficacy of the gene therapy, so that G+ cells are less likely to be infected than G- cells.
$\Gamma_{R5}(T, V_{R5})$<br>$\Gamma_{X4}(T, V_{X4})$	Terms modelling changes in infection rates with longer duration of infection and at lower total CD4+ T cell counts T. Here :<br>$\Gamma_{R5}(T, V_{R5}) = \left(1 + \eta_{R5} \int_0^t V_{R5}(\tau) d\tau\right)$<br>$\Gamma_{X4}(T, V_{X4}) =$<br>$= \left(1 + \eta_{X4} \int_0^t V_{X4}(\tau) d\tau\right)\left(\frac{1}{1 + e^{(g + \Delta g\, T)}}\right)$	$\eta_{R5} = 3.26 \times 10^{-13}$ /virion/day<br>$\eta_{X4} = 7.45 \times 10^{-14}$ /virion/day<br>$g = -114.13$<br>$\Delta g \in U[2.126 \times 10^{-9}, 5.95 \times 10^{-9}]$ /cells<br>where $U[2.126 \times 10^{-9}, 5.95 \times 10^{-9}]$ denotes a random number drawn from a uniform distribution with values ranging from 2.126×10^{-9} to 5.95×10^{-9}.	The term η_{R5} is selected so that viral accumulation during untreated infection results in R5 viral load increase from 4.5 to 5 log10 HIV RNA/mL over the 10 year period. The term η_{X4} is selected so that emergence of X4 on average accelerates AIDS by approximately 2-3 years. Parameters g and Δg are selected, so that during the course of untreated HIV infection, X4 virus emergence with a median time of 4 years post-PHI, with 5th and 95th percentiles of 1 and 8 years post PHI.
δ_I	Death rate of productively infected cells	$\delta_I = 0.66$ 1/day	Gives half-life for productively infected cells of approximately 1

			day [10,11].
p	Production rate of new virions from productively infected cells	$p = 320$ virions/cell/day	From [7,12].
c	Virion clearance rate from body	$c = 3$ 1/day	From [10].

References

1. Murray JM, Kaufmann GR, Hodgkin PD, Lewin SR, Kelleher AD, et al. (2003) Naive T cells are maintained by thymic output in early ages but by proliferation without phenotypic change after age twenty. Immunol Cell Biol 81: 487-495.
2. Vrisekoop N, den Braber I, de Boer AB, Ruiter AFC, Ackermans MT, et al. (2008) Sparse production but preferential incorporation of recently produced naïve T cells in the human peripheral pool. Proceedings of the National Academy of Sciences 105: 6115-6120.
3. McLean AR, Michie CA (1995) In vivo estimates of division and death rates of human T lymphocytes. Proc Natl Acad Sci U S A 92: 3707-3711.
4. Huenecke S, Behl M, Fadler C, Zimmermann SY, Bochennek K, et al. (2008) Age-matched lymphocyte subpopulation reference values in childhood and adolescence: application of exponential regression analysis. Eur J Haematol 80: 532-539.
5. Ahmed R, Gray D (1996) Immunological memory and protective immunity: understanding their relation. Science 272: 54-60.
6. Klein J (1990) Immunology. Blackwell Scientific : Boston.
7. Murray JM, Kaufmann G, Kelleher AD, Cooper DA (1998) A model of primary HIV-1 infection. Math Biosci 154: 57-85.
8. Bonyhadi ML, Rabin L, Salimi S, Brown DA, Kosek J, et al. (1993) HIV induces thymus depletion in vivo. Nature 363: 728-732.
9. Notermans DW, Pakker NG, Hamann D, Foudraine NA, Kauffmann RH, et al. (1999) Immune reconstitution after 2 years of successful potent antiretroviral therapy in previously untreated human immunodeficiency virus type 1-infected adults. J Infect Dis 180: 1050-1056.
10. Perelson AS, Neumann AU, Markowitz M, Leonard JM, Ho DD (1996) HIV-1 dynamics in vivo: virion clearance rate, infected cell life-span, and viral generation time. Science 271: 1582-1586.
11. Markowitz M, Louie M, Hurley A, Sun E, Di Mascio M, et al. (2003) A novel antiviral intervention results in more accurate assessment of human immunodeficiency virus type 1 replication dynamics and T-cell decay in vivo. J Virol 77: 5037-5038.
12. Haase AT, Henry K, Zupancic M, Sedgewick G, Faust RA, et al. (1996) Quantitative image analysis of HIV-1 infection in lymphoid tissue. Science 274: 985-989.
